# HIV-1 envelope facilitates the development of protease inhibitor resistance through acquiring mutations associated with viral entry and immune escape

**DOI:** 10.3389/fmicb.2024.1388729

**Published:** 2024-04-18

**Authors:** Ntombikhona F. Maphumulo, Michele L. Gordon

**Affiliations:** Department of Virology, Doris Duke Medical Research Institute, College of Health Sciences, University of KwaZulu-Natala, Durban, South Africa

**Keywords:** HIV-1, envelope, gag, PR, gp120, gp41, CXCR4, CCR5

## Abstract

**Introduction:**

There is increasing evidence supporting a role for HIV-1 envelope in the development of Protease Inhibitor drug resistance, and a recent report from our group suggested that Env mutations co-evolve with Gag-Protease mutations in the pathway to Lopinavir resistance. In this study, we investigated the effect of co-evolving Env mutations on virus function and structure.

**Methods:**

Co-receptor usage and n-linked glycosylation were investigated using Geno2Pheno as well as tools available at the Los Alamos sequence database. Molecular dynamics simulations were performed using Amber 18 and analyzed using Cpptraj, and molecular interactions were calculated using the Ring server.

**Results:**

The results showed that under Protease Inhibitor drug selection pressure, the envelope gene modulates viral entry by protecting the virus from antibody recognition through the increased length and number of N-glycosylation sites observed in V1/V2 and to some extent V5. Furthermore, gp120 mutations appear to modulate viral entry through a switch to the CXCR4 coreceptor, induced by higher charge in the V3 region and specific mutations at the coreceptor binding sites. In gp41, S534A formed a hydrogen bond with L602 found in the disulfide loop region between the Heptad Repeat 1 and Heptad Repeat 2 domains and could negatively affect the association of gp120-gp41 during viral entry. Lastly, P724Q/S formed both intermolecular and intramolecular interactions with residues within the Kennedy loop, a known epitope.

**Discussion:**

In conclusion, the results suggest that mutations in envelope during Protease Inhibitor treatment failure are related to immune escape and that S534A mutants could preferentially use the cell-to-cell route of infection.

## 1 Introduction

Protease Inhibitor (PI) treatment failure in the absence of mutations in the protease (PR) gene, that is not linked to treatment adherence, suggests that mutations outside of PR could contribute to PI resistance (Rabi et al., 2013). While mutations in gag have long been accepted to be associated with the development of PI resistance (Fun et al., 2012; Kletenkov et al., 2017; Su et al., 2019), Rabi et al. (2013) was the first to suggest that mutations in the HIV-1 envelope (Env) could also contribute to PI failure. They showed that *env* genes isolated from PI treatment failures were able to significantly reduce the activity of PIs (up to 10-fold) in the presence of wild-type PR and Gag (Rabi et al., 2013). More recent research has confirmed the involvement of Gag and gp41 during PI failure (Castain et al., 2019; Perrier et al., 2019; Hikichi et al., 2021). The possible co-evolution of the gag and *env* genes in the development of PI resistance has also been suggested, although very limited data is currently available (Maphumulo and Gordon, 2021).

HIV-1 Gag is a polyprotein made up of Matrix (MA), capsid (CA), nucleocapsid (NC), p6, and two spacer peptides p1 and p2 (Jacks et al., 1988), and cleavage of this polyprotein by HIV-1 PR is an important step during viral maturation (Pettit et al., 2005; Fun et al., 2012). Since Gag is the natural substrate for PR, mutations in gag have been associated with PI resistance (Dam et al., 2009; Clavel and Mammano, 2010) particularly those occurring at the cleavage sites. Mutations at non-cleavage sites have also been associated with PI resistance, albeit in a compensatory role, although many of these mutations have not yet been fully characterized, especially in non-B subtypes (Gatanaga et al., 2002; Myint et al., 2004; Özen et al., 2014). Previous studies from our group have identified several mutations in gag in HIV-1 subtype C (Q69K, R76K, Y79F, S111C/I, T239A/S, I256V, A431V, K436R, and P453L) that have been associated with LPV/r treatment failure (Singh, 2015; Marie and Gordon, 2019). Besides a few mutations found at the p7/p1 cleavage site, most other mutations occurred at non-cleavage sites in the MA and CA regions of Gag. The MA subunit is essential for targeting Gag to the cell membrane, and mutations found in this region (including R76K and Y79F) were previously suggested to enhance PR accessibility to the Gag cleavage sites (Parry et al., 2011; Su et al., 2019).

The HIV-1 *env* gene encodes a polyprotein (gp160) comprised of two glycoproteins that facilitate viral entry into target cells, gp120 and gp41. Gp120 binds to host cell surface receptors (CD4) and determines coreceptor (CCR5/CXCR4) specificity, while gp41 promotes fusion between viral and cellular membranes, releasing the viral nucleocapsid into the cytoplasm (Tedbury and Freed, 2014).

Researchers have reported mutations associated with PI treatment failure in both gp120 (Maphumulo and Gordon, 2021) and gp41 of Env (Coetzer et al., 2017; Manasa et al., 2017; Castain et al., 2019). Most reported gp120 mutations were located in the V1/V2 and V3 loop regions. The variable loops are known targets for neutralizing antibodies and are therefore the main structures responsible for immune escape (Pollakis et al., 2001; Yokoyama et al., 2016). This is mainly due to the high number of glycosylated sites, particularly in V1 and V2 (Leonard et al., 1990; Myers and Lenroot, 1992; Pollakis et al., 2001; Wang et al., 2013). In addition, these loops facilitate binding and specificity for the host cell coreceptors CCR5 and CXCR4. Pollakis et al., showed that the virus’ transition from an R5 to X4 phenotype was significantly influenced by the loss of an N-linked glycosylation site in the V3 region (Pollakis et al., 2001). Whether antiretroviral therapy (ART) provides the necessary conditions for the selection of X4 variants remains controversial, with reports supporting the hypothesis and those stating that the switch in co-receptor usage was independent of HAART (Delobel et al., 2005; Saracino et al., 2009). Nevertheless, a recent paper reported that the loss of R5 usage is a consequence of the co-evolution of the virus and the immune response, suggesting that mutations linked to evasion of the immune system could also evolve during drug selection pressure (Marichannegowda et al., 2023).

Env gp41 mutations associated with PI treatment failure that have been previously reported were found in the Ectodomain (I515L, S543A,T536M, A607T, D632E, and L641), Transmembrane (I688V), and cytoplasmic tail (CT) (L721I, P724S, I781T, T818, V832I, and I/L836F) (Coetzer et al., 2017; Manasa et al., 2017; Castain et al., 2019; Perrier et al., 2019; Maphumulo and Gordon, 2021). The N-terminal HR and C-terminal HR regions are located in an antiparallel orientation and are linked by a loop region that plays a critical role in fusion (Bär and Alizon, 2004). The CT of gp41 regulates both Env incorporation during budding, as well as fusion with the host cell during entry (Wyss et al., 2005; Viard et al., 2008).

Using Bayesian Network probability, Maphumulo suggested that Env mutations found in higher frequencies in LPV failures were linked to Gag mutations found in MA/CA (Q69K, R76K, S111C/I, T239S, I256V) and were involved in the pathway to LPV resistance (Maphumulo and Gordon, 2021). The MA domain and the CT tail of gp41 interact during incorporation of the Env glycoproteins into the budding virus (Yu et al., 1992; Freed and Martin, 1995), therefore it is quite possible that mutations in these regions could co-evolve as a result of PI selection pressure. CA plays an integral role not only in viral maturation, with the formation of the capsid core that encloses and protects the viral genome, but also in shielding the RT complex from restriction factors and facilitating trafficking to the nucleus and nuclear import during infection.

Structural studies have helped unravel how specific mutations contribute to PI resistance in PR, as well as, although to a lesser extent, the relationship between drug resistant PR and Gag (Codoñer et al., 2017; Marie and Gordon, 2019). A closer look at the impact of Env mutations on the structure of Env could also provide insights into the synergistic relationship between Gag and Env in the development of PI resistance. Therefore, in this study, previously identified Env mutations associated with PI failure were further characterized, and the effects of these mutations and related Gag mutations on the corresponding structures of Env (gp120 and gp41) and Gag were investigated.

## 2 Methodology

### 2.1 Sequences dataset

The impact of PI treatment on gp120, gp41, and Gag was investigated by comparing Env treated with naïve. Envelope sequences were obtained from 24 virologically failing PI-treated patients enrolled in the Protease Cleavage Site (PCS) study (2009–2013) at McCord and King Edward VIII hospitals Durban, South Africa (Marie and Gordon, 2019). All enrolled patients received Lopinavir/Ritonavir therapy for at least 6 months and had plasma HIV-1 RNA levels >1,000 copies/mL. In addition, sequences from 344 subtype C drug-naïve isolates were downloaded from the Los Alamos HIV-1 Database.^1^

### 2.2 Homology modeling

HIV-1 Env sequences obtained from six PI-treated isolates and one wild-type (WT) sample were initially modeled using the SWISS-MODEL web server.^2^ Template selection criteria were based on the best resolution and highest sequence identity. The crystal structure of HIV-1 concC_Base0 prefusion env trimer in complex with a human antibody fragment 3H109L and 35O25 variants at 3.5 Angstroms (6CK9) was used as a template for gp120. The crystal structure of the HIV-1 Env trimer 16,055 NFL TD CC (T569G) in complex with Fabs 35,022 and PGT124 (PDBID: 5UM8) was used to model the N-terminal regions of gp41. The membrane-proximal external region, transmembrane domain, and cytoplasmic tail were modeled using the PDBID: 7LOI as a template. The structure of the N-terminal 283-residue fragment of the HIV-1 Gag polyprotein (1L6N) was used as a template for Gag.

### 2.3 Molecular dynamic simulations

Molecular dynamics (MD) simulations were performed using AMBER 18, which was accessed at the Centre for High-Performance Computing.^3^ The GPU version of the Pmemd engine provided with the AMBER 18 package was used for MD simulation. The FF14SB Amber force field was used to describe the amino acid residues of the protein. Amino acid residues of the proteins were renumbered based on the dimeric form of the enzyme from 1 to 511. The systems were explicitly solvated by the TIP3P water molecules with a margin of 12.0 Å. Prior to equilibration, a two-step minimization was performed. Partial minimization of 2,000 steps with an applied restraint potential of 500 kcal/mol for both solutes were carried out, this was performed for 1,000 steps using the steepest descent method and then followed by 1,000 steps of conjugate gradients. Furthermore, full minimization of 1,000 steps were further performed by conjugate gradient algorithm without restraint. The whole system was then gradually heated from 0 K to 300 K, executed for 50 ps, in a manner that the systems maintained a fixed number of atoms and fixed volume. Also, the temperature was monitored using the Langevin thermostat with a collision frequency of 1.0 ps, with a potential harmonic restraint of 10 kcal/mol. After heating, the entire system was equilibrated at a constant temperature of 300 K, with the additional features such as several atoms and pressure also kept constant mimicking an isobaric-isothermal ensemble (NTP) as mentioned by Kehinde and coworkers, followed by an equilibration of each system estimating 500 ps. Finally, the Molecular dynamic simulations were performed for 100 ns, and for each simulation, the SHAKE algorithms were employed to constrict the bonds of hydrogen atoms. The simulations coincided with the NTP, with randomized seeding, the constant pressure-coupling of 1 bar maintained by the Berendsen barostat, a pressure-coupling constant of 2 ps, a temperature of 300 K, and Langevin thermostat with a collision frequency of 1.0 ps (Kehinde et al., 2019).

### 2.4 Post-dynamic analysis

The root mean square fluctuation (RMSF), root mean square deviation (RMSD), and radius of gyration (ROG) were performed using the CPPTRAJ modules implemented in Amber18. Snapshots were analyzed every 10,000 frames using CPPTRAJ. Ring server^4^ was used to identify hydrogen bonds and disulfide bridges. Origin data analysis software (Origin Lab, Northampton, MA) was used to generate all the graphs. Structures were viewed and analyzed in the UCSF Chimera software package^5^ (Pettersen et al., 2004).

### 2.5 N-linked glycosylation and loop length

We used online tools available at the Los Alamos sequence database to determine the characteristics of the HIV-1 variable regions (gp120- V1–V5 loops), including N-linked glycosylation sites, loop length, and the V3 loop net charge (NC).^6^ The Kruskal Wallis test in R-studio (version 4.3.1) was used to compare the differences between treated and naïve sequences.

### 2.6 Coreceptor usage

HIV-1 V3 sequences were submitted to Geno2Pheno[coreceptor]^7^ for classification of coreceptor usage (CCR5-using or CXCR4-using). A false-positive rate (FPR) cut-off of 2.5 and 20% was used (Poveda et al., 2012). In addition, the 11–25 rule was applied to define coreceptor tropism (Arif et al., 2017).

## 3 Results

### 3.1 The characteristics of gp120 and the effects of mutations in the structure

**Figure 1:**
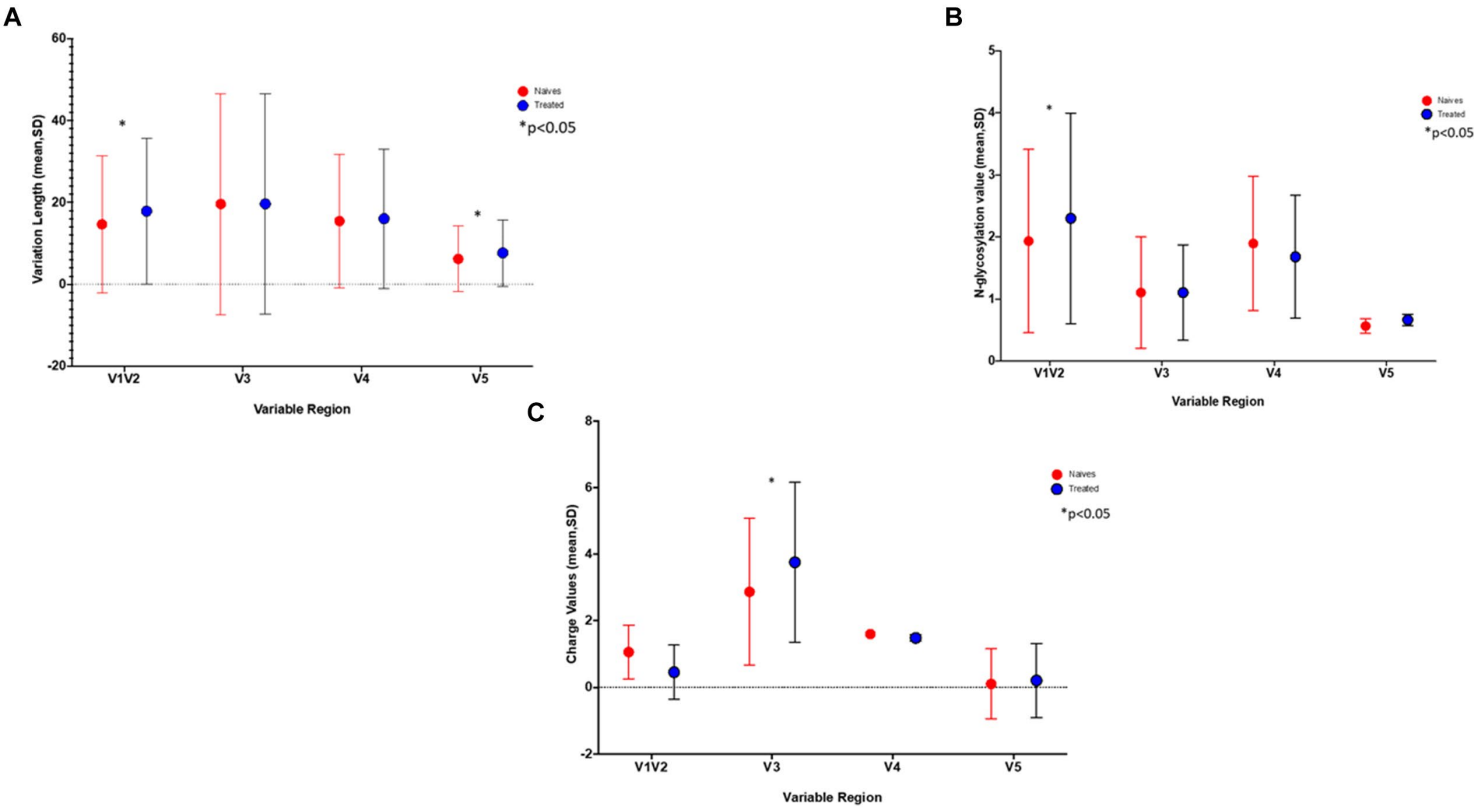
The graphs show the differences in **(A)** the length of the variable sites, **(B)** number of Glycosylation sites, and **C)** gp120 charge in treated (blue) versus naïve (red) sequences. *Indicates a significant difference found between the two groups (*p* < 0.05).

Differences in loop lengths were compared between the PI-treated and naïve groups. Using the Kruskal-Wallis test, the V1/V2 and V5 lengths were significantly longer in the treated group (*p*-values of 5.611e^−05^ and 2.2e^−15^ respectively), while the number of N- glycosylation sites for V1/V2 were also significantly higher (*p* = 0.008727) in the treated group (Figures 1A,B). Interestingly, putative glycosylation sites were not found in C5 and V5 (Supplementary Figure S1).

Putative phosphorylation sites that were seen in C1 and C3 (AA 110, 115, 264, and 365) usually used the SXXD/E motive while the variable regions (AA 161 and 303) used the TXXR/E motive. Of note, the S110N mutation resulted in the loss of this phosphorylation site which in turn resulted in the acquisition of an N-link glycan. Conserved N-myristoylation sites were mainly seen in C1 and C2.

### 3.2 Coreceptor prediction

Using a 20% FPR, 37.5% of the treated group were predicted to be CXCR4 viruses and this decreased to 20.8% when using an FPR of 2.5% (Table 1), this was still significantly higher than the 1.2% obtained in the naïve group (*p* = 0.0002). A similar result was obtained when using the 11/25 rule. The V3 charge was significantly higher in the treated versus naïve group (*p* = 0.015) (Figure 1C).

**Table 1:** Coreceptor prediction in subtype C sequences, using a FPR of 20 and 2.5%.

	Coreceptor prediction		
Sample ID	Predicted coreceptor (20% FPR)	Predicted coreceptor (2.5% FPR)	11/25 rule tropism
PCSK18	R5	R5	R5
PCSK19	R5	R5	R5
PCSK20	R5	R5	R5
PCSK22	R5	R5	R5
PCSK24	X4	R5	R5
PCSK28	R5	R5	R5
PCSK33	R5	R5	R5
PCSK36	X4	X4	X4
PCSK59	R5	R5	R5
PCSK61	R5	R5	R5
PCSK70	R5	R5	R5
PCSK75	X4	R5	R5
PCSK83	X4	X4	X4
PCSK84	X4	R5	X4
PCSK89	X4	X4	X4
PCSK90	X4	X4	R5
PCSK93	R5	R5	R5
PCSK108	R5	R5	R5
PCSK114	R5	R5	R5
PCSK120	R5	R5	R5
PCSK128	R5	R5	R5
PCSK141	X4	X4	X4
PCSK145	R5	R5	R5
PCSK153	X4	R5	R5

### 3.3 Effect of potential PI-associated resistance mutations on the structure of gp120

**Figure 2:**
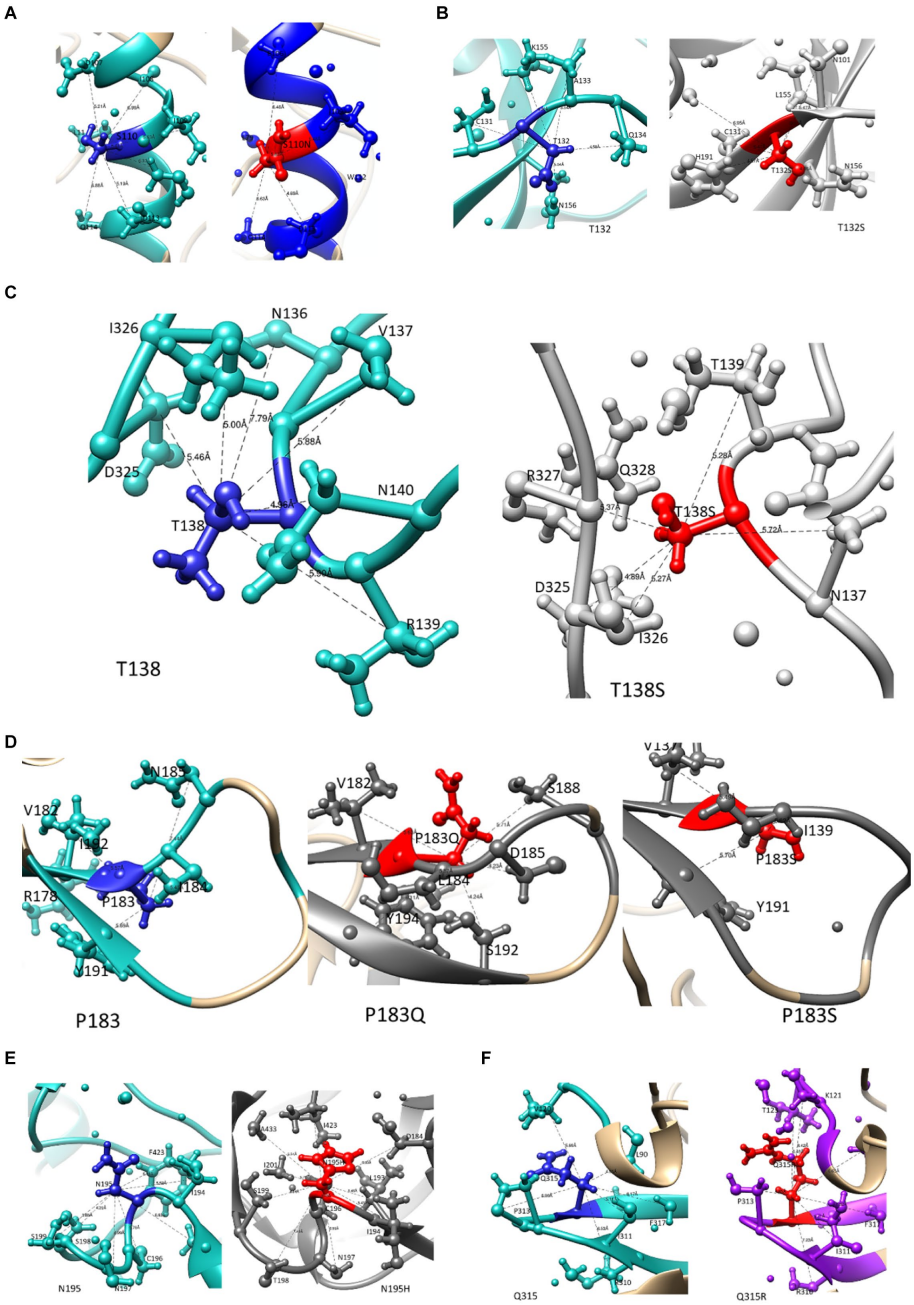
The interaction of mutations that potentially contribute to PI failure with other AA residues within a 5 angstrom radius. WT residues are highlighted in blue, and mutations are presented in red ball and stick for **(A)** S110N in the C1 region, **(B)** T132S and **(C)** T138S in the V1 region, **(D)** P183S and **(E)** N195H in the V2 region, and **(F)** Q315R in the V3 region.

A previous study from our laboratory using a Bayesian Network analysis reported mutations in Env that potentially contribute to PI failure (C1-S110N; V1-T132S, T138S; V2-P183S; V2- N195H and V3-Q315R). Figures 2A–D illustrates how these mutations affect Env structure. We also provide information on their interactions with other residues within a 5 Å-radius range (Supplementary Table S1), as well as the number and distance of hydrogen bonds. The WT Serine at position 110 interacts with positions E106—S115 and R429, while the mutant S110N interacts with the same positions except R429. Position 429 in the C4 region forms part of the surface of gp120 that makes contact with the CD4 coreceptor. T132 and T132S interact with similar AAs, whereas, WT T132 showed additional interactions with S158, N186, and D187 while T132S interacts with additional AAs V134 and Y191 as shown in Figure 2B. Although mutation T138S and WT T138 interact with the same AAs, T138S interacted with additional AAs (N141, G142, N143, N152, E153, R327, and Q328). P183, P183Q, and P183S also interact with similar AAs, P183Q showed additional interaction with N186 and S189, whereas P183S showed additional interaction with N186G, Y193, and I194. Furthermore, only P183S contains the hydrogen bond interaction with D185. Both N195 and mutation N195H interact with AAs at the coreceptor-specific site and CD4 contact residues in V2 and C4, respectively, and shows hydrogen bond interaction with S198 and S199. In the 5 Å zone, both Q315 and Q315R are shown to interact with the same positions (V120, K121, L122, T163, I309, R310, I311, G312, P313, G314, A316, and F317). In addition, Q315R interacted with T123 and P124 in C1. Furthermore, they both form two hydrogen bonds with I311, while mutant Q315R forms an additional hydrogen bond with K121 (2.92 Å) found at the coreceptor binding site.

### 3.4 Gp41 interactions

**Figure 3:**
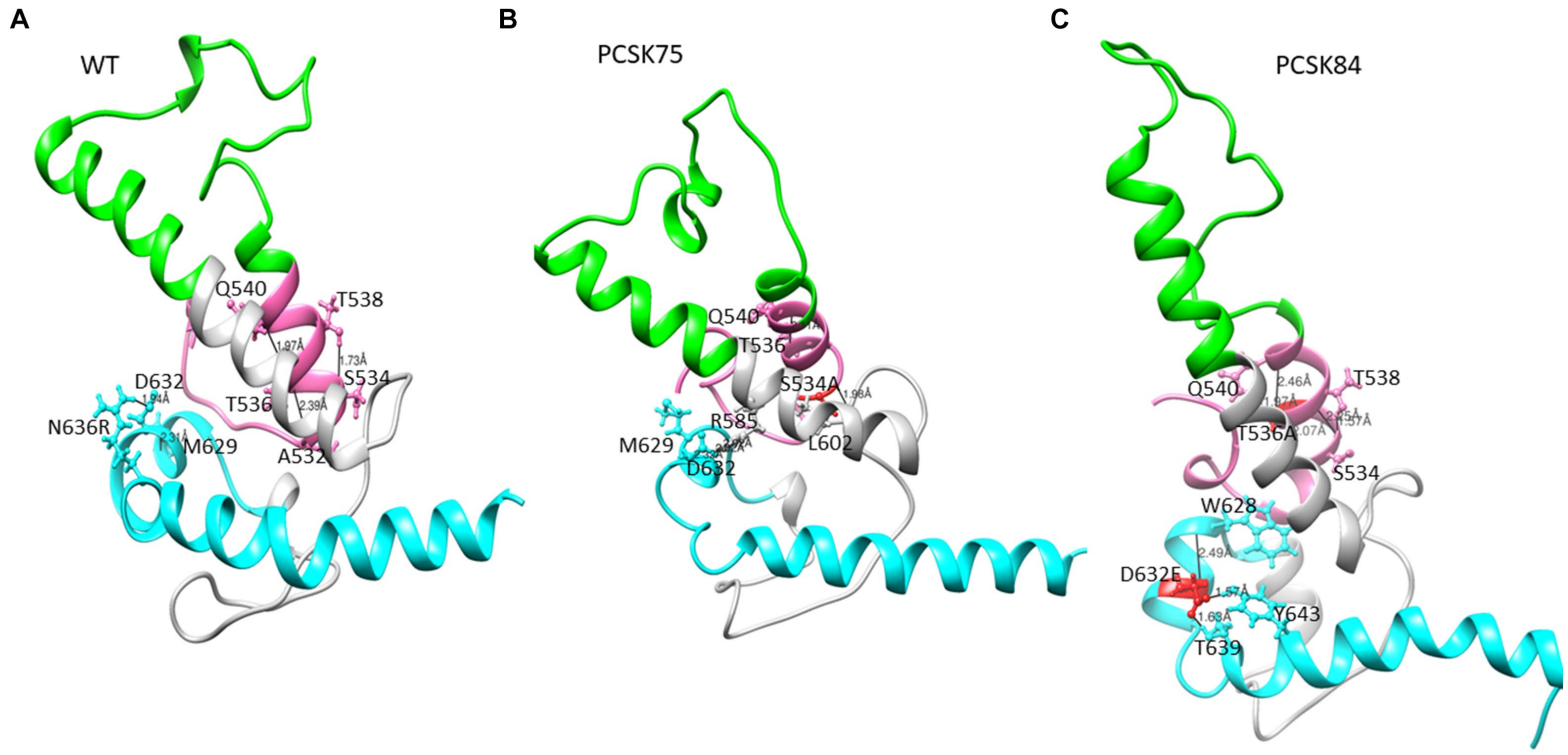
Part of the ectodomain region showing the **(A)** WT structure residues S534 and T536 interacting with residues T538, A532 and Q540, and D632 interacting with M629 and N636R respectively, **(B)** PCSK75 showing S534A interacting with residues within the FPPR and in the loop region, and **(C)** PCSK84 showing T536A interacting with residues within the FPPR and D632E located in HR2 interacting with residues within the HR2. The colour presentation is: FP (pink), HR1 (green), and HR2 (cyan). Mutations are shown in red ball and stick.

**Figure 4:**
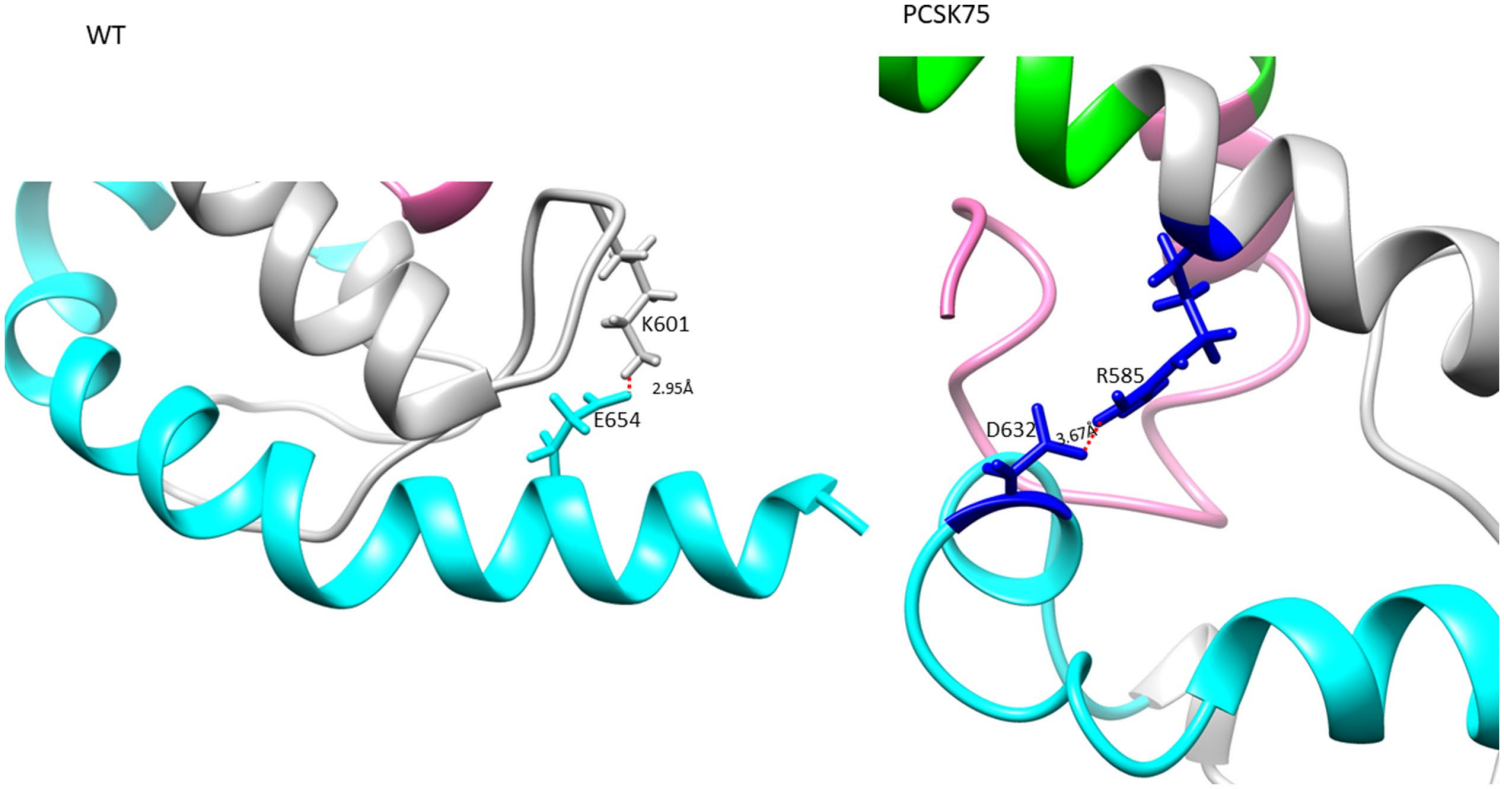
The Ectodomain structure showing a salt bridge between residues K601 and E654 in the WT, and PCSK75 showing a salt bridge between D632 and R585. Grey indicates the loop region, cyan is HR2, and pink is the FP region.

**Figure 5:**
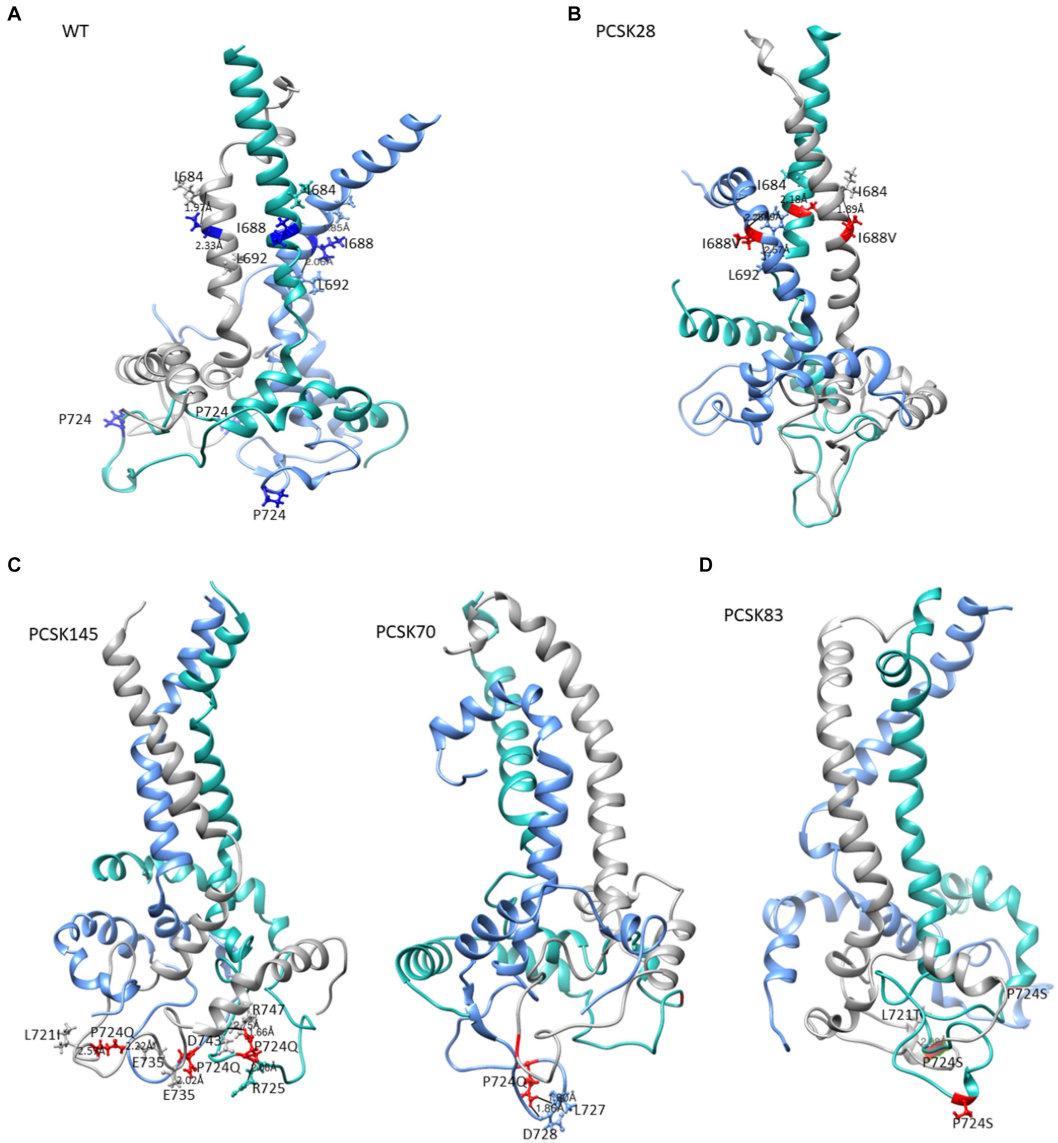
The structure of gp41 presenting MPER in purple, TM in blue, CT in green. Mutations are shown in red ball and stick. The structure of **(A)** the WT, **(B)** PCSK28 showing the TM mutation I688V interacting with MPER residues, **(C)** PCSK70 and PCSK145 structures showing the P724Q mutation with no interaction and **(D)** PCSK83 structure showing the P724S mutation.

Representative structures containing the gp41 mutations previously identified using BN analysis are shown below. Due to the availability of template structures for the C-terminal region of gp41, the modeled structures were divided into the ectodomain (Figures 3, 4) and the TM/CT (Figure 5). Hydrogen bond interactions from both the Ring server and Chimera are shown in Supplementary Table S2 and their interactions with other residues within a 5-radius range shown in Supplementary Table S4.

From the Ring Server and Chimera, S534A formed a hydrogen bond with M530 (also within the FPPR) in one sample, with L602 in the loop region in a second sample and with both M530 and L602 in a third sample, Figure 4 shows how the S534A mutation caused the hydrogen bond formed between the WT S534 and L537 and T538 in the FPPR (shown in the pink α-helix, WT; Figure 2A) to shift to L602 at the beginning of the loop region (shown in gray). Furthermore, S534A did not share a hydrogen bond with T536 that was seen between the WT A532 and T536. The hydrogen bonds seen between the WT T536 and A532 and Q540 in 4/5 of the samples remained the same in the T536A mutant.

All 4 samples with D632E formed hydrogen bonds with W628 (in combination with R585, N636D, M629, T639, and Y643), which was also seen with D632, which formed hydrogen bonds with W628, R629, and N639R. Of note, D632 in HR2 formed an ionic interaction with R585 in the loop region in 5/15 samples. Interestingly, those with D632E and that did not have any PI resistance mutations in PR (*n* = 3/4) also did not show this ionic interaction. Another ionic interaction was seen between K601 in the loop region and E654 in HR2 In the WT sample that was not structure (Figure 5B). seen in any of the mutant samples (Figure 4). In both samples with I688V (found in the α-helix of the TM region), hydrogen bonds were formed with I684, F685, and L692; these hydrogen bonds were also seen in the WT.

Lastly, P724Q/S occurred in a loop region in the CT (Figure 5C). P724Q formed hydrogen bonds with L721I, L727, and D728, and intermolecular interactions were also seen between P724Q of chain 1 and E735, D743, and R747 of chain 3. In addition, P724Q interacted with L721I/T in 3/5 samples, of these three samples, two further interact with D743, two of five samples interact with L727 and D728 and the last interaction was with E735 in different chains; P724S interacted with L721T, and one sample interacted with E725 in different chain. WT P724 did not form any hydrogen bonds with other residues.

### 3.5 Gag mutations associated with PI-failure

**Figure 6:**
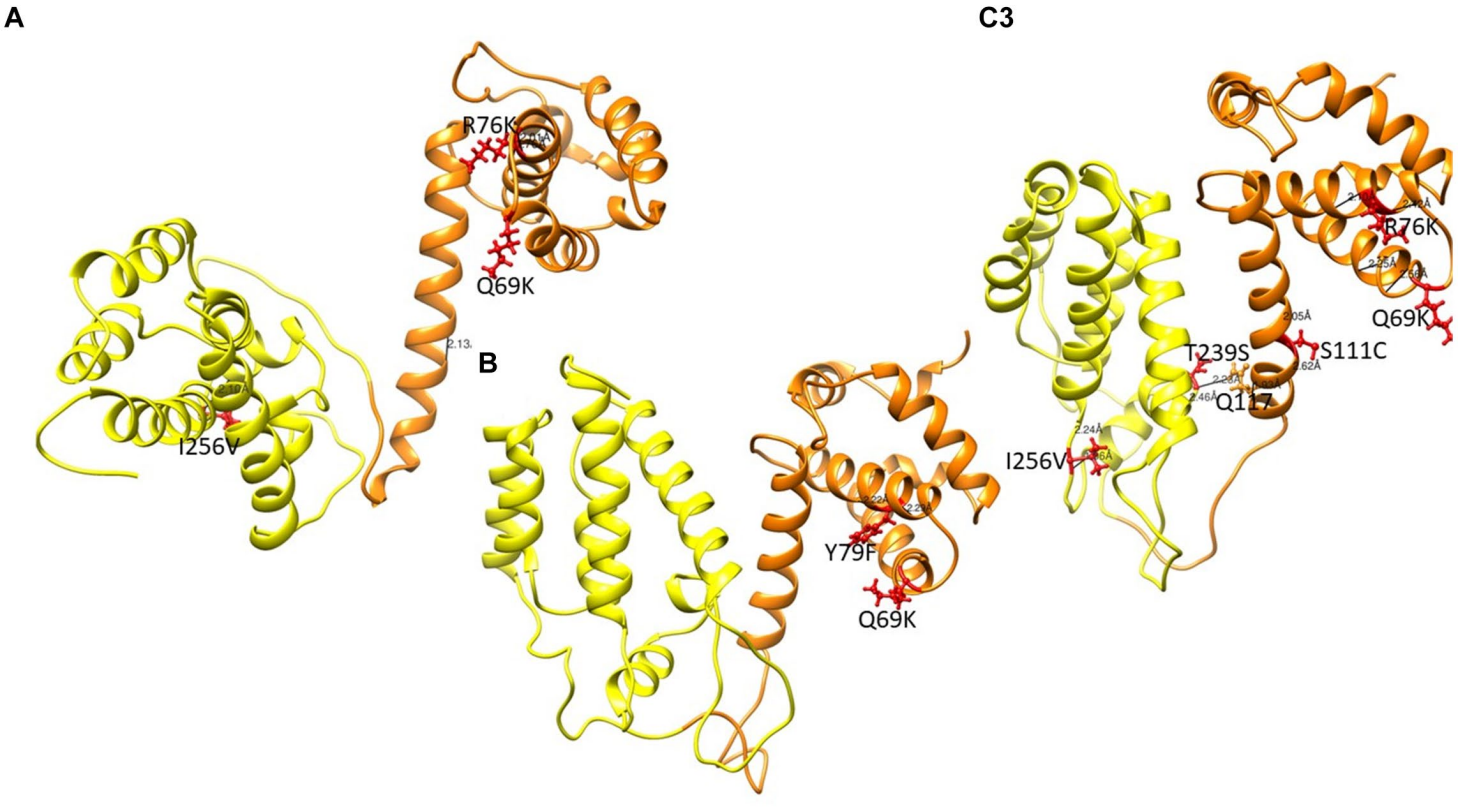
Gag structure presenting the MA and CA in orange and yellow respectively. Mutations are shown in red ball and stick. **(A)** PCSK70 structure showing Q69K, R76K and I256V within gag **(B)** PCSK84 structure showing Q69K and Y79F. Both patients did not harbour mutations in the protease gene. **(C)** PCSK108 showing Q69K, R76K and I256V, as well as S111C and T239s. This patient harboured both PR and gag mutations.

Structures of MA and CA showing mutations previously linked to PI failure and Env are shown in Figure 6. Interestingly, most MA and CA mutations occurred in the alpha-helices. Of note, the wild type Q69 and mutant Q69K formed hydrogen bonds with residue Q65 and residue Q65 and P66, respectively. Mutants with either R76K and S111C lost a hydrogen bond, while Y79F and I256V mutants did not have any change in hydrogen bonding compared to the WT. However, the length of the bond for the WT-I256 and mutant-I256V are 3.086 Å and 2.062 Å, respectively. Interestingly, in mutants with Q69K R76K and Y79F rarely occurred in the same isolate. Mutants with T239S in the CA domain formed a hydrogen bond with Q117 in the MA domain as shown in Supplementary Table S4.

## 4 Discussion

In this study we saw an increase in the number of N-glycosylation sites in the V1/V2 region, increased sequence variation in V1/V2 and V5, as well as an increased V1/V2 loop length in the treated group vs. naïve group. Taken together, since these types of changes have previously been shown to protect the virus against neutralization by reducing antibody binding affinity through steric hindrance, this suggests that evasion of the host immune system is also one of the mechanisms used by the virus during the development of drug resistance (Pollakis et al., 2001; van Gils et al., 2011; Guo et al., 2012; Wang et al., 2013). Specifically, the length of the V2 and V5 regions seen in our naïve group (34–41 amino acids and 10–12 amino acid respectively) were similar to those reported by Mandizvo et al. (2022) for untreated HIV-1 subptype C (34–48 and 10–13 respectively) (Mandizvo et al., 2022), which strongly supports that the increased V2 and V5 length (41–55 and 10–16 respectively) in our treated group could be related to treatment.

Switching co-receptor usage could also be another way that the drug-resistant virus facilitates infection. Both P183S/Q and N195H in V2 occurred at CCR5-specific binding sites, and a change from asparagine at N195 has been shown to reduce CCR5 binding (Thielen et al., 2010). In fact, 38% (9/24) of the treated sequences were predicted to be CXCR4 viruses, and of those with N195H (6/9) four were predicted to be CXCR4 viruses. Similarly, those with the V3-Q315R mutation found at the tip of the V3 loop were also predicted to be CXCR4 coreceptor using viruses. The evidence for a coreceptor switch in the treated group was strengthened by the significantly higher charge in V3 (*p*-value = 0.015) in the treated group vs. naïve group. A closer look at the effects of these mutations on the Env structure, particularly N195H in the V2 region showed a change to the opening of the coreceptor binding site. Of note, the CXCR4 predicted viruses that also harbored the N195H mutation showed structural alterations in the GPGR tip, which could impact the β-turn and hence co-receptor engagement at that site (Pastore et al., 2006).

In gp41, key mutations that were positively selected in PI-treated isolates were seen in the FPPR (S534A and T536A), which is an important region involved in the association of gp120 with gp41 during viral fusion (Freed et al., 1990; Cao et al., 1993). Lu et al. (2021) showed that S534 formed hydrogen bonds with N656 in HR2 and suggested that this interaction stabilized the formation of the Env trimer, and was crucial for viral fusion (Markosyan et al., 2003; Lu et al., 2019, 2021). While we did not see any interaction between S534 and N656 in the wildtype isolates, possibly because this study was limited to the interactions seen in the monomer structures, the mutant S534A formed a hydrogen bond with L602, found in the disulfide loop that connects HR1 and HR2. The disulfide loop is at the site of non-covalent contact with C5 in gp120 and forms part of the furin recognition site where gp120-gp41 are cleaved (Cao et al., 1993; Caffrey, 2001; Sen et al., 2007; Yi et al., 2016). It is therefore possible that S534A and T536A could negatively affect the association of gp120-gp41 during viral entry and suggests a shift to a cell-to-cell route of infection in the presence of these mutations (Freed et al., 1990; Lu et al., 2019, 2021; Hikichi et al., 2021). Hikichi et al. (2021) showed that where viral entry is inhibited, the virus reverts to a predominant cell-to-cell mode of infection (Hikichi et al., 2021). This suggests that they will preferentially use the cell-to-cell route of infection in the presence of these mutations.

Analysis of the impact of D632E on Env structure showed an intramolecular salt bridge between 632 and 585 that was also seen in the wildtype structures. A salt bridge between D632 and K574 was previously reported, and was associated with viral entry, inhibition and the stabilization of the interaction between the α-helixes of HR1 and HR2 (Jiang and Debnath, 2000; He et al., 2008). It is possible that this shift in the salt bridge could be responsible for the same function, although this needs further investigation. Interestingly, sequences with D632E that did not harbor any PI associated resistance mutations, did not form the salt bridge, however they did maintain the same hydrogen bonds (D632E with W628 and N636) as the WT.

I688V also did not appear to have a major effect on the structure, although the distance between I688V and I584 in the MPER region was shorter than WT, whereas distance between I688V and L692 within the TM was longer than the WT. The effect of P724Q/S, on the other hand, was more apparent, with the gain of hydrogen bonds in the trimer that were absent in the WT P724. P724Q/S formed both intramolecular and intermolecular hydrogen bonds with L721A/T, L727, D728, and E735; and D743, respectively. Intramolecular interactions have been shown to contribute to stability of the hydrophilic loop, whereas intermolecular interactions have been associated with strengthening of the trimer interface (Eastep et al., 2021).

From the structural changes observed in Gag, the Q69K that was previously reported to coevolve with gp120 and minor protease mutations (Codoñer et al., 2017; Maphumulo and Gordon, 2021), showed a longer α-4/5 loop than the WT. This position has been reported to contribute to the structural flexibility of MA and the longer loop seen in the mutant suggests that it occupied more volume than the WT (Khan and Vihinen, 2007). R76K and S111C both lost a hydrogen bond compared to the WT, also possibly contributing to an increased flexibility within the α-4/5 helices, respectively. Previous studies have reported a loss of hydrogen bonds to be associated with flexibility around the helix which allows better access of the solvent to the carbonyl group, thereby increasing the affinity or accessibility of MA-CA cleavage sites for PR (Woolfson and Williams, 1990; Parry et al., 2011; Fun et al., 2012).

Effects of the other Gag mutations under investigation were more related to structural stability, such as the gain of a hydrogen bond between T239S in CA and Q117 in MA, that could contribute to the stability of the MA-CA structure, and I256V that showed a shorter distance to W249. It has been suggested that a stronger interaction in α-helix structure could influence viral assembly and maturation (Fun et al., 2012; Saito and Yamashita, 2021).

A limitation of the study was that the effect of mutations on the structure of Gag and env could only be shown separately as it was difficult to model the whole genome. Furthermore, this study did not conduct functional assays to confirm the effect of Env mutations, either alone or in combination with Gag mutations, on viral fitness and PI efficacy. However, following future studies will be done using the same data:

I. Replication capacity and drug susceptibility.
II. viral entry through immune escape and coreceptor switching.
III. Investigate the effect of Gag and Env mutations on HIV-1 assembly and budding during PI failures.

In conclusion, our results proposes that gp120 indirectly facilitates PI resistance through acquiring mutations that increase the length of V1/V2, consequently increasing the overall number of N-glycosylation sites, as well as increasing the overall charge in V3. When taken together, these results suggest that gp120 mutations modulate viral entry activity by (i) Protecting the HIV-1 from antibody recognition and leading to immune escape, (ii) coreceptor switching which could possibly enhance viral replication, suggesting an interplay between immune escape and the development of drug resistance. In addition, mutations in the gp41 Kennedy epitope are more related to immune escape. Moreover, it has been demonstrated that the gp41 mutations reported here affect the flexibility of the protein. The S534A mutation could influence fusion by interacting with the gp120-gp41 interface, and this suggests that viruses under drug selection pressure will preferentially use a cell-to-cell route of infection in the presence of these mutations.

## Data availability statement

The original contributions presented in the study are included in the article/Supplementary material, further inquiries can be directed to the corresponding author.

## Ethics statement

The studies involving humans were approved by the Biomedical Research Ethics Committee (BREC). The studies were conducted in accordance with the local legislation and institutional requirements. Written informed consent for participation was not required from the participants or the participants’ legal guardians/next of kin in accordance with the national legislation and institutional requirements.

## Author contributions

NM: Conceptualization, Data curation, Formal analysis, Funding acquisition, Investigation, Methodology, Project administration, Resources, Software, Validation, Visualization, Writing – original draft. MG: Conceptualization, Resources, Software, Supervision, Validation, Visualization, Writing – review & editing.

## Footnotes

^1^ http://hiv-web.lanl.gov

^2^ https://swissmodel.expasy.org

^3^ http://www.chpc.ac.za

^4^ https://ring.biocomputingup.it/

^5^ https://www.rbvi.ucsf.edu/chimera

^6^ https://www.hiv.lanl.gov/content/sequence/VAR_REG_CHAR/index.html

^7^ https://coreceptor.geno2pheno.org/
