# Supplementary material for: HIV-1 envelope facilitates the development of protease inhibitor resistance through acquiring mutations associated with viral entry and immune escape
Source: Front Microbiol. 2024 Apr 18;15:1388729. doi: 10.3389/fmicb.2024.1388729 (PMC11063367; doi:10.3389/fmicb.2024.1388729)
Supplement: Supplementary file 1 [file Presentation_1.pdf]

Putative Sites

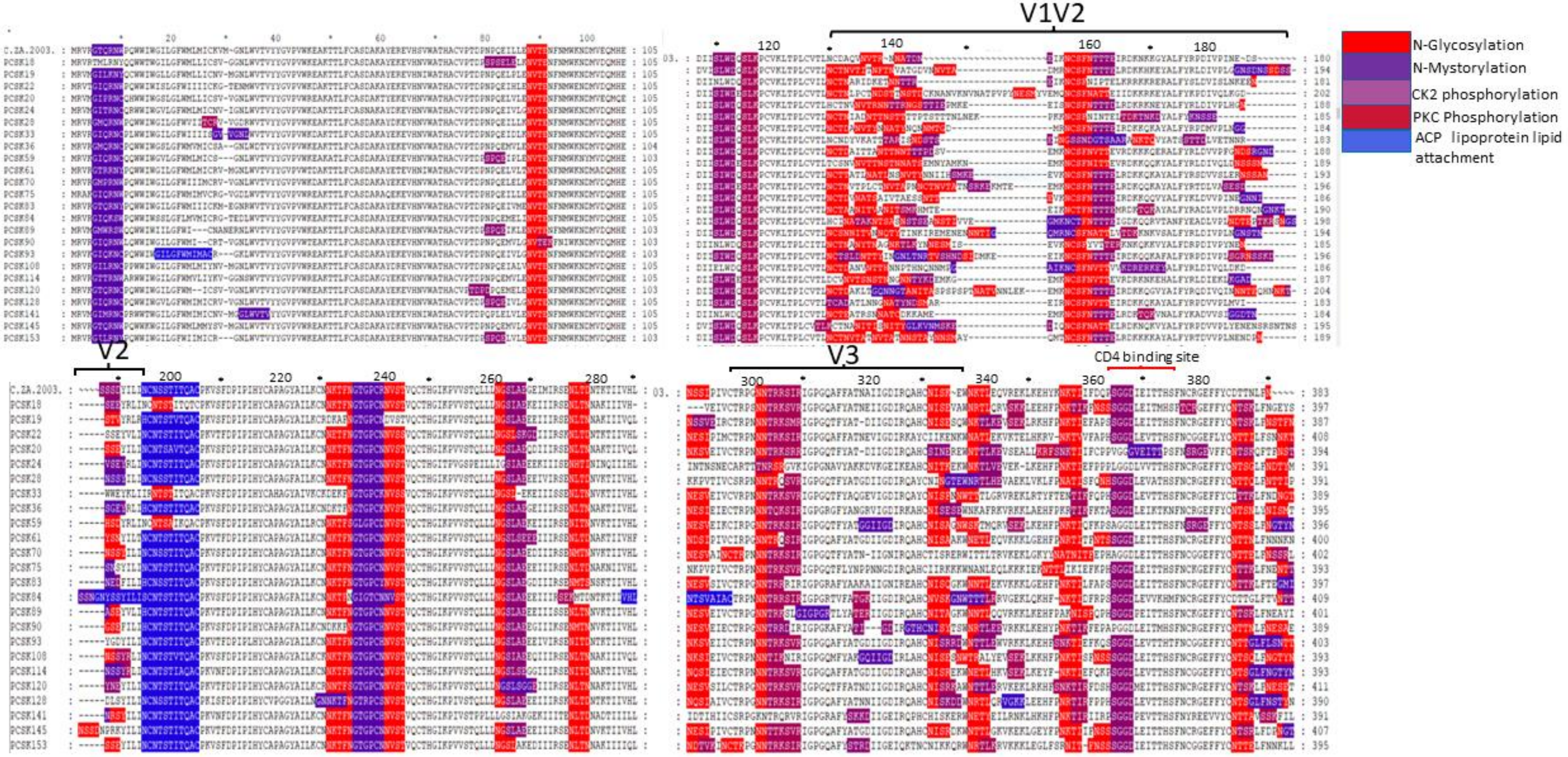

Supplementary Figure 1: Alignment of the subtype C PI-treated sequence. Putative sites were determined using PROSITE in GENEDOC**.** Functional sites are shown by the colored boxes: red - N-glycosylation site; purple - myristoylation site; plum – CK2 phosphorylation magenta - PKC phosphorylation; blue - ACP-lipoprotein lipid attached site.

Supplementary Table S1: HIV-1 WT and the mutations that potentially contribute to PI failure, and their interaction with other gp120 AA residues within a 5-radius range.

					AA interaction within 5Å							
**S110**	**S110N**	**T132**	**T132S**	**T138**	**T138S**	**P183**	**P183Q**	**P183S**	**N195**	**N195H**	**Q315**	**Q315R**
E106	E106	N130	T130	N136	S136	V181	I181	V181	D180	D180	V120	V120
D107	D107	C131	C131	V137	N137	V182	V182	V182	L193	L193	K121	K121
I108	I108	D133	N133	R139	T139	F184	L184	I184	I194	I194	L122	L122
I109	I109	M154	V134	N140	N140	G185	D185	G185	N195	C196	T163	T123
L111	L111	K155	M154	G324	N141	D187	N186	G186	C196	N197	I309	P124
W112	W112	N156	K155	D325	G142	S188	S187	D187	N197	T198	R310	T163
D113	D113	C157	N156	I326	N143	E190	S188	T188	T198	S199	I311	I309
Q114	Q114	S158	C157		N152	Y191	S189	N190	S199	T200	G312	R310
S115	S115	N186	H188		E153	R192	D190	S192	I201	I201	P313	I311
R429		D187	S189		D325		Y191	Y193	K421	I423	G314	G312
		S188	Y191		I326		R192	I194	I423	H425	A316	P313
		G189			R327				N425	Q428	F317	G314
					Q328				Q428	R429		G316
									G429	G431		F317
									G431	R432		
									A433	A433		

Supplementary Table 2: The gp41 mutations and the residues they interact with through hydrogen bonds. The hydrogen bonds distance taken at the threshold of less than, or equal to 3.5Å. Mutations that have PR mutations are highlighted in bold.

PID	Mutations	H-bond (3.5Å)	H-bond in Chimera
WT	S534	L537 & T538	T538
	T536	A533	A532 & Q540
	D632	W628 & N636R	M629 & N636R
	I688	I684, F685, G691 & L692	I684, G691 & L692
	P724	NONE	NONE
PCSK75	S534A	L602	G531 & L602
PCSK93	D632E	NONE	W628 & T639
PCSK36	P724Q	L721T, R744 & R761	L721T, R729, D743, R744 & R761
PCSK70	P724Q	P727L & D728	P727L & D728
PCSK145	P724Q	E734, E735 & R747	L721I, R725, E734, E735, D743 & R747
PCSK24	S534A	M530	M530 & G600
	D632E	W628 & N636D	W628
PCSK28	T536A	A532, A533 & Q540	A532 & Q540
	I688V	I684 & F685	I684, F685 & L692
PCSK83	T536A	Q540	A533 & V539A
	P724S	L721T	L721T
PCSK84	T536A	A532, A533, V539 & Q540	A532 & Q540
	D632E	N636E & Y643	W628, T639 & Y643
PCSK114	T536A	NONE	A532, V539A & Q540
	P724S	E735 & E736	P722 & E736
PCSK120	D632E	W628	W628 & R633
	P724Q	L727, D728, L730 & E731	G726, L727, D728 & L730
**PCSK33**	**D632E**	**R585, W628 & M629**	**R585 & W628**
**PCSK61**	**S534A**	**M530 & L602**	**L602**
**PCSK89**	**T536M**	**V539 & Q540**	**Q540**
	**P724Q**	**S716 & L721A**	**L721A**
**PCSK108**	**T536A**	**A525, A532, V539 & Q540**	**A532 & Q540**
	**I688V**	**I684, F685, L692, G693 & L694**	**I684 & L692**

Supplementary Table 3: The Gag mutations, WT, and the residues they interact with within a 5-radius range.

				AA interaction within 5Å						
S534	S534A	T536	T536A	D632	D632E	I688	I688V	P724	P724Q	P724S
M530	T529	A525V	A525V	T627	T627	L684I	L684I	L720	T721	T719
G531	M530	A532	A532	W628	W628	F685	F685	T721	P722	L720
A532	G531	A533	A533	M629	M629	I686	I686	P722	N723	T721
A533	A532	S534	S534	Q630	Q630	M687	M687	N723	R725	P722
V535	A533	V535	V535	W631	W631	V689	V689	R725	G726	N723
T536	V535	L537	L537	K633	K633	G690	G690	G726	P727	R725
L537	T536	T538	T538	E634	E634	G691	G691	P727	D728	G726
I603	L537	V539	V539	I635	I635	L692	L692	D728	R729	P727
W623	T538	Q540	Q540	E636	E636				P730	D728
W628	L593			T639					I733	
	G600									
	K601									
	L602									
	I603									
	W628									

Supplementary Table 4: The Gag mutations and the residues they interact with through hydrogen bonds and VDW bonds. Taken at the distance threshold of less or equal to 3.5Å for hydrogen bond and the distance threshold for VDW is 0.5Å. Mutations with PR mutations are shown in bold.

PID	Mutations	H-bond (3.5Å)	VDW (0.5Å)
WT	Q69	Q65	
	R76	G71, T72, E73, F79, N80	
	Y79	V82, A83	
	S111	E107, Q108, K114, T115	
	T239	S234	
	I256	W249	
PCSK18	Q69K	Q65, S66, Q196, D200	Q65
	R76K	E71, T72, E73, Y79F, N80	E73, N80, E207
	Y79F	L75, R76K, V82, A83	
	S111I	G107, Q108, A115	E107
	I256V	W249	W249
PCSK24	Y79F	A83	Q65, L68, L75
PCSK75	Q69K	P66	
	I256V	W249	
PCSK84	Q69K		
	Y79F	V82, L75, A83	Q65, L68, L101
PCSK93	R76K	T72, E73, N80	E73
	S111C	E107, Q108, L114, A115T	
PCSK70	R76K	T72, E73, N80	G71
	I256V	W249	P231, W249
PCSK128	Q69K	P66S	
	I256V	W249	
PCSK59	Q69K		T240
	R76K	T72, E73, N80	
	S111C	T115	
PCSK83	R76K	G71,Q69, T72,F79, N80	N80
	Y79F	L75, R76K, V82, A83	L101, L68
	S111C	E107	W249
PCSK114	I256V	W249	
**PCSK19**	**I256V**	**P252, W260, R259**	**V251, W260**
**PCSK33**	**Q69K**		
	**R76K**	**T72, E73, Y79, N80**	**G71, N80**
	**I256V**	**W249**	
**PCSK61**	**Q69K**		
	**R76K**	**E72, N80**	
	**I256V**	**W249**	**I261**
**PCSK89**	**R76K**	**S72, Y79, N80**	
	**S111C**	**E107**	
**PCSK90**	**Q69K**	**E65, P66**	
**PCSK108**	**Q69K**		
	**R76K**	**G71, T72, E73, N80**	
	**S111C**	**E107, A115M**	
	**T239S**	**Q117, S234**	
	**I256V**	**W249, I261**	
**PCSK153**	**Q69K**	**L283**	**I282**
	**R76K**	**T72, E73, Y79F, N80**	**E73**
	**Y79F**	**L75, R76K, V82, A83**	**L101**
	**I256V**	**W249**	
